# Predictive effect of resilience on self-efficacy during the COVID-19 pandemic: The moderating role of creativity

**DOI:** 10.3389/fpsyt.2022.1066759

**Published:** 2022-12-22

**Authors:** Yanhua Xu, Guang Yang, Chongshan Yan, Jiatong Li, Jingwei Zhang

**Affiliations:** ^1^School of Geography and Environment, Jiangxi Normal University, Nanchang, China; ^2^College of Teacher Education, Capital Normal University, Beijing, China

**Keywords:** resilience, self-efficacy, creativity, college students, COVID-19

## Abstract

**Introduction:**

To appraise the relationship and mechanism between resilience and self-efficacy in the context of the COVID-19 pandemic, we proposed a model to investigate the effect of resilience on self-efficacy and the moderating effect of creativity in this regard.

**Methods:**

Scales that measured resilience, creative behavior, and self-efficacy were rated by 881 college students in China to establish the moderating model.

**Results:**

The results showed that resilience and self-efficacy of participating college students were positively correlated, which meant that high resilience could predict a high level of self-efficacy. Moderating analysis using the SPSS PROCESS plug-in showed that creativity was an important element of resilience that positively affected self-efficacy and that this moderating effect was more significant in participants with a high degree of creativity.

**Discussion:**

These findings can provide a better understanding of the relationship between resilience and self-efficacy in demonstrating the traumatic impact of the COVID-19 pandemic on adolescent mental health and academic performance.

## 1 Introduction

A recent threat to the health of people worldwide is the ongoing outbreak of the respiratory disease known as COVID-19 (1). To date, the world has paid a huge price during this pandemic in terms of the loss of human life and economic impact (2). To contain the epidemic, many countries have imposed social distancing and declared lockdowns to regulate movement control (3). In such circumstances, many people are obliged to use the social media among the limited means of communication. Thus, national television channels or social media platforms have been adopted for education (4). One damaging aspect of social media use lies in its potential to spread false, alarmist, and exaggerated information that can create fear, stress, depression, and anxiety in people with and without underlying mental illness (5). In the case of the COVID-19 pandemic, college students may experience varying degrees of fear, stress, depression, and other emotions because their learning is restricted to a few social and online channels; this can profoundly affect their mental health and academic performance (6). Accordingly, we started with examining psychological resilience to evaluate whether it was related to college students’ self-efficacy under the epidemic environment, and how creativity and active thinking might play a regulating role in this respect.

The global epidemic of the coronavirus disease presents a major threat to public health worldwide (7). There have been considerable differences in the way people cope with this crisis. The capacity to withstand setbacks, adapt positively, and recover from adversity is collectively known as *resilience* (8). Resilience is essential for coping effectively with difficulties, uncertainties, and changes (9), and it can be applied in settings related to prevention (pre-exposure to stress) or treatment (recovery from the adverse effects of such anxiety) (10). Past research has shown that resilience can counteract the negative effects of poor health (11) and reduce mortality by 6% (12), as well as moderate depression (13) and negative emotions (14). However, few studies have used resilience as an influencing variable to examine its mechanism of action in college-student populations during the COVID-19 pandemic. Here, we delve into how resilience has affected other psychological factors among college students during the pandemic. At the same time, given the challenges posed by the disease outbreak, the role of creativity cannot be underestimated. Beghetto and Kaufman propose the 4C model, which can be used to analyze creativity in that context (15). The category of *MINI-C* describes exploratory behaviors that are not necessarily considered novel when viewed in the larger historical perspective, but that have personal meaning to the individual creator. *LITTLE-C*, or everyday creativities, encompasses the ordinary creative behaviors of most persons. *PRO-C* is reserved for those who have reached or are approaching an expert level of creativity, even if they may not have achieved excellence. The medical staff on the front line who attend to the pandemic patients and the technical experts who develop platforms for online education to protect students show evidence of PRO-C. Those categorized under *BIG-C* are often considered geniuses in their field and represent the pinnacle of what is possible (16). During the COVID-19 pandemic, people have tended to develop new forms of amusement and entertainment because of isolation or the reduction of in-person entertainment. Such behavior can be seen as the concentrated expression of MINI-C and LITTLE-C. On January 11, 2020, the genetic sequence of the COVID-2 coronavirus, was released, triggering intensive scientific activities to develop a vaccine for this disease (17). Therapies targeting the immunopathology of infection then became a major focus, in addition to approaches that targeted the virus directly or block its infection (18). These activities require BIG-C. Creativity, as a means of coping with the uncertainty caused by the pandemic and meeting personal needs in this environment can enable people to find meaning in the mundane (19). However, there is not too much research on the mechanism of creativity as a variable that moderates psychological and behavioral change among college students. Therefore, we decided to look into how creativity might moderate the relationship between behavioral variables among students at the beginning stages of the COVID-19 pandemic.

In the relationship between creativity and self-efficacy, the latter is sometimes conceptualized as a component of resilience and post-traumatic growth (20–22), which suggests the importance of resilience for self-efficacy. Other factors that are also related to self-efficacy include autonomy (23), multiple health behaviors (24), multiple chronic diseases (25), various parenting styles (26), and adolescent adaptation to traumatic experiences (27). Nevertheless, few studies have focused on the complex relationship between resilience and self-efficacy, between resilience and creativity, or the possible inter-relationships of resilience, self-efficacy, and creativity.

## 2 Theoretical background

### 2.1 Psychological resilience

A starting point for studying the concept of resilience is with the recognition that a certain proportion of young people today are not overwhelmed when faced with serious trials and difficulties (28). Resilience has been defined as the interaction of psychological traits in the context of stressful processes (29) to shield the individual from negative effects (30). In previous studies of resilience, a number of protective factors have been identified, viz. tolerance (31), positive emotions (32), extroversion (33), self-efficacy (34), spirituality (35), self-esteem (36), and positive influence (37). These findings also support Rutter’s view that resilience is an interactive concept involving a combination of serious risk experiences that nonetheless end with relatively positive psychological outcomes (38).

The definition of psychological resilience is still being discussed in the academic community. For example, it has been defined as a class of phenomena characterized by good outcomes in the face of serious threats to adaptation or development [(39), p. 228]. In other words, it reflects personal qualities that facilitate one to thrive in the face of adversity [(40), p. 76], while encompassing a complex repertoire of behavioral tendencies [(41), p. 197]. An individual’s degree of success in demonstrating resilience in confronting major challenges can be conceptualized as an interplay of factors panning out in a manner that is either beneficial or detrimental to his or her wellbeing (42, 43). Traits of young people who exhibit resilience have included a “relaxed temperament” [(44), p. 185], optimism (45, 46), personal determination and perseverance (46–48), as well as family cohesion (49).

Several theoretical models of resilience exist. For example, in a study of resilience in old age, an overarching construct of resilience has been postulated that explains the function of several psychological resources (self-esteem, and personal competence and control) (50). A three-part model of resilience (encompassing environment, physical behavior, and cognition) was used to demonstrate that social support, adaptive health practices, adaptive coping, and optimism were important in helping police officers face adversity (51). Hence, a preliminary cognitive model of resilience can potentially facilitate the application of cognitive approaches to the study of resilience in adversity (52).

### 2.2 Self-efficacy

According to the theory of social cognition and self-efficacy, one’s beliefs about one’s abilities and the results of one’s efforts can have a powerful influence on one’s behavior (53, 54). The core of the self-efficacy theory is that the initiation and persistence of behaviors and actions are primarily determined by judgments and expectations about behavioral skills and abilities and the likelihood of successful coping with environmental demands and challenges (55). Self-efficacy has received much attention in educational research. For example, it was shown that perceived self-efficacy for learning was correlated positively with students’ ability in arithmetic. Generally, students with high self-efficacy are given the opportunity to be engaged in different types of tasks (56). Those who are confident in their academic abilities are more effective at monitoring their own work, solve problems more efficiently, and exhibit greater persistence than their peers who are equally able but have lower self-efficacy (57). They also work harder, assess their progress more frequently, and engage themselves in more self-regulatory strategies to succeed further (58). One’s beliefs about his or her self-efficacy can be influenced by emotional and physiological states, such as anxiety, stress, fatigue, and mood; for example, high levels of anxiety undermine self-efficacy (59).

Currently, when studying self-efficacy, most researchers use an adapted version of the self-efficacy scale developed by Lent et al. (60), which was originally designed to assess the sources of mathematical self-efficacy in college students. It has since been adapted for use in academic and social settings (61–64). Matsui et al. also de-signed a scale to measure the sources of mathematical self-efficacy in college students (65), and this scale has also been used with middle school students (66). In addition, Hampton developed the Source of Academic Self-Efficacy Scale, which was subsequently validated and applied to students with learning disabilities (67).

A previous study showed that, empirically speaking, resilience was closely related to self-efficacy (68). Another study also found that resilience was moderately to highly associated with components of self-efficacy (69). Given these findings, it is likely that resilience and self-efficacy are positively correlated, which leads to the following hypothesis:

**Hypothesis 1:** *Psychological resilience is a positive predictor of self-efficacy.*

### 2.3 Creativity

Research in creativity had its roots in the mid-twentieth century when differences in creativity between disciplines began to be studied in the 1930s (70–72). Since the term creativity was put forward, people have discussed its connotation and theory; the basis of creativity has been constantly enriched and expanded (73, 74). Guilford views creativity, in its narrow sense, as an individual’s ability to perform creative acts to a noteworthy extent (75). Stein argues that it is necessary to distinguish between the internal and external frames of reference of creativity (76). Creativity is seen as the ability to produce work that is both novel (that is, original and unexpected) and appropriate (that is, useful and adapted to specific tasks) (77–81). Collins postulates that motivation stemming from personal engagement is essential to a high level of creativity in any field (82). At the individual level, creativity is closely related to personal life; for example, creativity is used when one tries to solve a difficult problem at work or in daily life (83). An analysis of the content of the *Journal of Creative Behavior* shows creativity enhancement and education to be the most common themes (84).

In addition to the 4Cs model, previous studies offer the following theories and models of creativity. The first is the 4Ps model which integrates people, process, products, and the environment (19). The second is the componential model of creativity that encompasses cognitive, personal, motivational, and social factors, including domain-related skills, creativity-related skills and task motivation (85). The model was subsequently modified by the addition of a social context component (86). The third is the creativity-investment theory, which posits that intelligence, knowledge, way of thinking, personal traits, motivation, and environment all affect creativity; here, creativity is seen as the comprehensive effects of individual psychological mechanisms and environmental factors (80).

All of the above theories align broadly with the focus of this study, namely the relationship between resilience and creativity in college students. As mentioned earlier, resilience is a positive attribute that determines the individual’s response to stress and adversity. In reviewing the literature, we found that stress and creativity have been explored extensively. For example, artists and other creative professionals may find it difficult to first establish and then to maintain themselves continually in creative work (87–89). In the current post-Fordist conditions, creative abilities are constantly challenged (90, 91). Stability, the opposite of innovation, has been much maligned by business writers and consultants despite its distinct benefits for individuals and society (92). However, stress, time constraints, and social pressure related to their work can be powerful levers that enhance the effectiveness of creative problem-solving methods (93). In addition, several studies on the relationship between perception (or emotion) and creativity (94–96) have found that positive emotions are sometimes positively associated with creativity (97, 98). Given that resilience is a positive emotion and attitude generated in the face of hardship and stress, these studies contribute to the understanding of the relationships between resilience and creativity, as well as among hardship, stress, and creativity. So, we hypothesized that:

**Hypothesis 2:** *Psychological resilience has a positive predictive effect on creativity.*

Several articles in recent literature examine the connection between creativity and self-efficacy. As with other forms of behavior, creative expressions can be influenced by one’s self-judgments about his or her ability to produce novel and useful results. Such self-judgments, also called creative self-efficacy (98), are an important extension of the general concept of self-efficacy (99). Proximity to a mastery level of performance, belief in their ability to innovate, and teacher feedback have been positively correlated with the creative self-efficacy of students (100). Investigating this attribute in students may help support educators’ and researchers’ long-standing efforts to enhance creativity (54, 101–103). Self-efficacy provides the motivation to initiate creative behavior. Individuals are much more likely to engage in a task if they assume they will have a successful outcome. The motivation to succeed is high under such circumstances (104). Bandura also suggests the possibility of a relationship between creative behavior and self-efficacy (105). Based on this, we hypothesized that:

**Hypothesis 3:** *Creativity has a positive predictive effect on self-efficacy.*

There are several reasons why creativity can moderate the relationship between resilience and self-efficacy. First, creativity and its manifestation through unique art-making or problem-solving abilities have long been connected with divergent thinking (106). Metz posits that the ability to think creatively predicts resilience (107). Second, Bandura argues that innovation requires an unshakable sense of efficacy to persevere creatively; due to the involvement of high risks and multiple obstacles, one needs adequate self-efficacy to persist in creative work. Creative self-efficacy is also a necessary precursor of creative efforts (108). Although studies have explored the mechanisms underlying the inter-relationships between creativity, resilience, and self-efficacy, few have explored the moderating role of creativity in these realationships. On this theoretical and empirical basis, the following hypothesis is proposed:

**Hypothesis 4:** *Creativity plays a moderating role in the relationship between psychological resilience and self-efficacy.*

**FIGURE 1:**
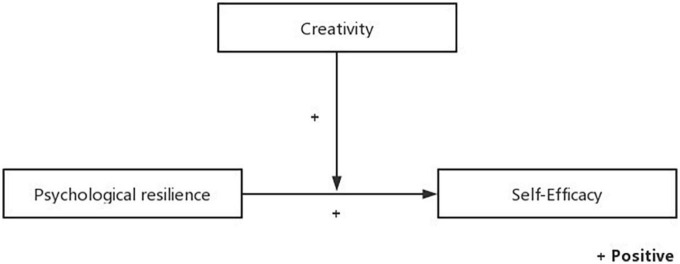
Conceptual model of the hypotheses.

The hypothetical model is shown in Figure 1.

## 3 Materials and methods

### 3.1 Participants and procedures

For this study, 918 participants were recruited from a polytechnical college in Guangdong Province, China, that had more than 20,000 full-time students. From them, we selected 19–21-year-olds as the survey respondents. The number was eventually reduced to 881 qualified participants, 317 males (35.982%) and 564 females (64.018%). Before the study design was finalized, we conducted exploratory focus-group interviews with the students to investigate their emotional characteristics and psychological state. Most interviewees said that they had experienced depression during the COVID-19 pandemic.

This study used a related design. We collected data through an online questionnaire that participants completed between April 10 and June 15, 2020. During the recess of an online course, consenting participants scanned a QR code that took them to the online questionnaire. (QR or Quick Response is a readable barcode that can be scanned with a mobile phone tablet or other device equipped with a camera to reach a specific link. In China, QR codes are widely used to for such functions as financial payment, verification of identity, and querying information). The purpose and utility of this study had earlier been introduced to the students in detail to ensure that they were participating on a voluntary basis.

### 3.2 Materials

The questionnaire used in this study consists of four parts: demographic information and three scales totaling 60 items that measured resilience, creativity, and self-efficacy. The scales used to measure resilience and general self-efficacy, originally developed in English, were translated into Chinese for this study. In order to improve the quality of the translation, we adopted the back-translation method: The first researcher translated the English text into Chinese, then the second researcher translated the Chinese text back into English. A third researcher compared the original version, the translated version, and the back-translated version of the scales to determine the accuracy of translation Before finalizing the questionnaire, the translated content was revised and optimized to ensure the equivalence of the scale. All questionnaires took a total of 10–15 min to complete.

#### 3.2.1 Resilience scale for chinese adolescents

After reviewing relevant studies from Chinese and foreign sources, we selected the Resilience Scale for Chinese Adolescents (109). The scale has 27 items divided into five dimensions: Goal focus, emotional control, positive cognition, family support, and interpersonal assistance. Respondents are asked to rank the degree of their agreement with each statement on a five-point Likert scale ranging from one (totally disagree) to five (totally agree). For example, “I have a clear purpose in my life,” “I have difficulty controlling unpleasant emotions,” “I have a lot of mood swings and tend to have big ups and downs,” and “I think adversity is motivating.” In the study, the scale had a Cronbach’s alpha of 0.860.

#### 3.2.2 General self-efficacy scale

A Chinese-language version of the General Self-Efficacy Scale was used in this study (110). The original scale contains 10 items. Combined with the specific situation of students and research needs, we removed the last three items after a group discussion, reducing the number of items to seven. The scale is rated on a four-point scale (one = completely 1 incorrect, four = completely correct). For example, “I can always solve problems if I try,” “I am confident that I can cope effectively with anything that comes my way,” and “I am able to face difficulties calmly because I trust my ability to deal with problems.” In this study, the internal consistency coefficient of the scale was 0.875.

#### 3.2.3 Runco ideational behavior scale

A Chinese-language version of the Runco Ideational Behavior Scale was used in this study to measure creative ideation (111). The scale consists of 23 self-report items that measure the level of creative behaviors in daily life, using a five-point rating (one = strongly disagree, five = strongly agree). For example, “I have a lot of novel ideas,” “I can come up with ideas or solutions that no one else has thought of,” and” I’m good at combining ideas in ways that no one else has tried before.” In this study, the scale had an internal consistency coefficient of 0.938.

### 3.3 Analysis of data

SPSS 26.0 was used for data processing and analysis in this study. To ensure the validity of these self-reported data, Harman’s single factor test (112) was used to check for common method biases before data processing. A total of 57 items in the questionnaire related to the three variables were tested. The results showed 10 factors with eigenvalues greater than 1. The contribution rate of the 10 factors to the total variance was 61.821%, and the explanation rate of the first factor was only 23.403%, which did not reach the critical standard of 40% (113). Hence, there was no significant common methodological bias in this study.

We next performed descriptive analysis, correlation analysis and model testing on the data based on the study hypotheses. First, we examined data centralization and dispersion. Then, we calculated Pearson’s correlation coefficients to test the relationships among the independent, dependent, and moderating variables. Using these results, we further investigated the research hypotheses. and used the SPSS PROCESS (version 4.0) plug-in to test the moderating effect of the model. (The PROCESS plugin was developed by Hayes. specifically for path-analysis–based moderation and mediation analysis and their combinations).

## 4 Results

### 4.1 Descriptive statistics and correlation analysis

Descriptive statistics and Pearson product moment correlation coefficients were calculated using SPSS 26.0. The results of the analysis are shown in Tables 1, 2.

**TABLE 1:** Descriptive statistics for the three variables.

Variable	*N*	*M*	*SD*
Psychological resilience	881	3.4173	0.4431
Male	317	3.4524	0.4396
Female	564	3.3975	0.4447
Self-efficacy	881	2.3180	0.5370
Male	317	2.3074	0.5516
Female	564	2.3239	0.5295
Creativity	881	3.2487	0.5633
Male	317	3.2515	0.5602
Female	564	3.2472	0.5660

**TABLE 2:** Correlations among variables.

S. no	Variables	1	2
1.	Psychological resilience		
2.	Self-efficacy	0.2660***	
3.	Creativity	0.1980***	0.4750***

*N* = 881. ***p < 0.001.

It was found that resilience was positively correlated with creativity (*r* = 0.198, *p* < 0.001) and self-efficacy (*r* = 0.266, *p* < 0.001). The creativity of the participants was positively correlated with their self-efficacy (*r* = 0.4750, *p* < 0.001). Therefore, the results of correlation analysis provided preliminary support for the subsequent test of the moderating effect.

### 4.2 The moderating analysis of creativity

We used Model 1 of the SPSS PROCESS plug-in to perform the multiple regression analysis, with resilience as the independent variable, self-efficacy as the dependent variable, and creativity as the mediating variable. As shown in Table 3, resilience was significantly correlated with self-efficacy (β = 0.2159, *SE* = 0.0358, *p* < 0.001), indicating that resilience had a significant impact on self-efficacy i.e., a higher level of resilience among college students predicted a stronger sense of self-efficacy. Creativity was significantly correlated with self-efficacy (β = 0.4333, *SE* = 0.0286, *p* < 0.001); thus, creativity could significantly predict self-efficacy. The interaction of resilience and creativity was significant (β = 0.1698, *SE* = 0.0603, *p* < 0.01), meaning that while resilience had an impact on self-efficacy, creativity also had an impact on self-efficacy at different levels. To investigate further, we used the bootstrap method to determine that the confidence intervals (at 95% confidence) for the interaction item between resilience and creativity on self-efficacy, [0.0515, 0.2881] did not contain a zero value. Therefore, the moderating model of psychological resilience and self-efficacy was established, with creativity as the moderating variable between psychological resilience and self-efficacy.

**TABLE 3:** Analysis of the moderating effect of creativity on self-efficacy.

Predictors	Self-efficacy					
	β	*SE*	*t*	*p*	95% CI (Lower)	95% CI (Upper)
*Resilience*	0.2159	0.0358	6.027***	0.0000	0.1456	0.2863
*Creativity*	0.4333	0.0286	15.1394***	0.0000	0.3771	0.4895
Resilience*creativity	0.1698	0.0603	2.8175**	0.0049	0.0515	0.2881
*R* ^2^	0.2635					
*F*	104.5958***					

*N* = 881. **p < 0.01; ***p < 0.001.

**FIGURE 2:**
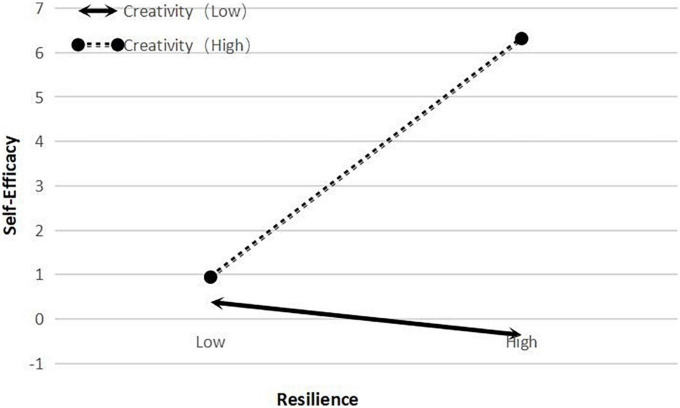
The relationship between resilience and self-efficacy for high and low scores for creativity.

**FIGURE 3:**
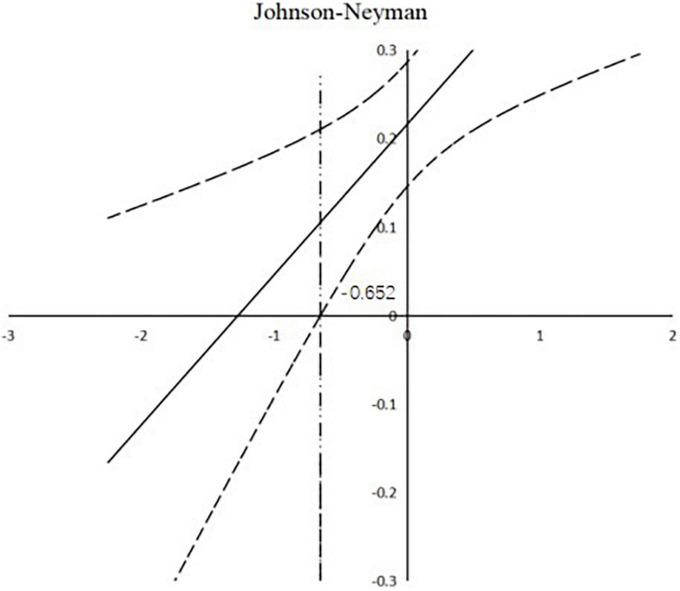
The relationship between resilience and self-efficacy for high and low scores for creativity, using the Johnson-Neyman technique (114).

To further analyze the moderating effect of creativity, we divided the scores for creativity score into a low group (M − 1 SD) and a high group (M + 1 SD) before performing a simple slope analysis (see Table 4). The results showed that the 95% confidence intervals did not include a zero value, and that creativity affected the strength of the relationship between self-efficacy and creativity. When the score for creativity was high (M + 1 SD), creativity was a stronger predictor of self-efficacy (see Figures 2, 3). When the value of creativity is greater than –0.652, the moderating effect shows a significant state and plays a positive role. At the same time, it can be seen from Figure 3 that the moderating effects of creativity on resilience and self-efficacy have no negative effect, but only change the size of the influence.

**TABLE 4:** Conditional effects at specific levels of creativity.

**Score for creativity**	**Estimate**	***SE***	***t***	***p***	**95%CI**	
					**Lower**	**Upper**
*− 1 SD*	0.1202	0.0498	2.4153*	0.0159	0.0225	0.2179
*M*	0.2159	0.0358	6.027***	0.0000	0.1456	0.2863
+ *1 SD*	0.3117	0.0490	6.3649***	0.0000	0.2156	0.4078

*N* = 881. *p < 0.05; ***p < 0.001.

## 5 Discussion

### 5.1 Summary of findings

In this study, we developed a moderating model to explore the relationship between resilience and self-efficacy among students during the COVID-19 pandemic, using a sample of 881 college students from a polytechnical college in Guangdong, China. We also investigated the potential moderating impact of creativity in this relationship. The results showed that (1) resilience was positively correlated with self-efficacy and creativity; (2) creativity was positively correlated with self-efficacy; (3) the interaction of resilience and creativity was positively correlated with self-efficacy, indicating that creativity had a significant moderating effect on resilience and self-efficacy. Specifically, when the creativity scores of participants were high, this effect was more significant.

These findings provide further evidence of the relationship between resilience and self-efficacy. They also demonstrate how resilience and creativity can enhance self-efficacy of college student following traumatic events during the COVID-19 pandemic. These results are in agreement with the hypotheses proposed in this study and in previous studies.

At this juncture, we appraise an overview of the above findings.

First of all, the results of this study are basically consistent with H1 and those appearing in previous studies, namely that there is a positive correlation between psychological resilience and self-efficacy (115, 116). Since a positive link can be expected between mental toughness and self-efficacy, adolescents may experience more self-efficacy during the COVID-19 pandemic if they have strong resilience. In a study of patients with diabetes-caused foot ulcers, factors such as self-efficacy were significantly higher for the high-resilience group than for the low-resilience group (117). Self-efficacy among college students enrolled in online courses was reflected in the completion of their courses, social and academic interactions with classmates, use of the course management system, and interaction with instructors (118). Resilience can be a key factor in the psychosocial care of patients (119), and college students who experience excessive negative emotions due to the COVID-19 pandemic can improve their self-efficacy through online learning platforms that promote adaptive development.

Secondly, the results of this study are in agreement with those of other similar studies: psychological resilience has a significant impact on creativity (120). In other words, people with mental toughness are likely to be more creative. When one examines the concepts of creativity and resilience, certain associations can be inferred (121). When one has strong mental toughness, his or her problem-solving stamina and resistance to stress are at relatively higher levels, thus promoting creativity. The individual’s personality has an important influence on creativity, expressed mainly in his or her resilience in the face of obstacles and other imitations of courage and determination. A correlation between the social component of mental toughness and creativity can hence be inferred, with the former supported in the social environment and in social relationships that reinforce the involvement of creative activities.

The large number of natural and man-made disasters makes it imperative to confront and respond to crisis and trauma. In the social sciences, psychological resilience is understood as “the process of bending and bouncing back from adversity” (74). Similarly, creativity is essential for adaptation, adjustment, or problem solving (122). Contradictory traits. namely optimism vs. realism, logic vs. naivety, introversion vs. extroversion, can be defining characteristics of creative personalities (123, 124). A living mind links resilience and creativity, with people having strong resilience possessing the flexibility to adapt to specific situations; this flexibility allows them to express creativity. The findings in the present study imply that college students with strong resilience have the ability to respond to specific circumstances associated with the pandemic and they can recover from difficult situations through flexible thinking that reflects creativity. Their psychological resilience contributes to the molding of their personality, which in turn facilitates development of creativity. This may be more evident in the development of MINI-Cs, and the career Cs needed for future development. Thus, creative social engagement and psychological resilience support each other (125, 126).

Thirdly, these results are consistent with previous studies showing that, in line with H3, creativity has a positive effect on self-efficacy. As Bandura mentions, successful experiences increase self-efficacy; substitution or imitation also affects one’s self-efficacy, while direct experience or alternative experience based on one’s persuasion to oneself can increase one’s self-efficacy (53). Successful experiences, alternative experiences and imitation, and verbal persuasion need to be novel and practical in order to have an impact in this respect. Bandura hence contends that there is a significant correlation between the generation of creativity and discovery on the one hand, and strong self-efficacy on the other (53). Accordingly, the epiphanies required for these idea generation processes may be important in enhancing self-efficacies that arise in different domains. Self-efficacy is perceived and defined differently in different fields. The concept of “creative self-efficacy” refers to the self-evaluation of individuals when they are engaged in a specific task; in other words, it is their confidence in their ability to invent new products (99). The impact of creativity self-efficacy has been noted in entrepreneurship (127), small-business performance (128), employee performance (129), and other domains. Therefore, it would be possible for creative college students to develop creative self-efficacy whereby they can strive to overcome difficulties experienced during the pandemic, and be confident and optimistic about themselves.

The COVID-19 pandemic had negatively impacted many components of students’ development, including their predisposition to stress, anxiety, and depression. They experienced many negative emotions arising from rumors and uncertainties that led to a decrease in their self-efficacy. However, those with positive creativity were able to counter these tendencies to engender positive experiences of success, alternative experiences, and imitation and verbal persuasion, which in turn, enhanced their self-efficacy. In this regard, creativity reflects tolerance and responsiveness to new environmental stimuli. Individuals with high creativity may be more sensitive and responsive to pandemic-related events and react more positively.

Fourthly, the results from the present study corroborate H4: Creativity can mediate the relationship between resilience and self-efficacy. High resilience traits predict more creative thinking (130) and in the present study, self-efficacy was better predicted when creativity was higher (i.e., M + 1 SD) levels of creativity. Creative thinkers are more likely to change their minds and use multiple methods to solve problems instead of giving up, thus further improving their psychological resilience (131). Entrepreneurial activity is accelerated by higher self-efficacy, where the capacity to do a job creatively is fundamental to the individual’s self-definition (132). Notwithstanding this, creativity can also play a negative role if creative thinking is too out-of-step with reality and practicality. In this connection, college students may be unable to adapt to certain pandemic situations, resulting in self-doubt and learned helplessness, traits which are not conducive to self-efficacy.

In this study, it can be seen that creativity moderates the relationship between mental toughness and self-efficacy to some extent. It hence plays a very important role in the relationship between psychological resilience and self-efficacy.

### 5.2 Theoretical contribution and practical significance

This study provides fresh insights into the psychological state of Chinese college students in the context of the COVID-19 pandemic. Previous studies tend to regard creativity as a dependent variable, leading to the testing the influence of psychological variables in different situations on creativity. In our study, creativity is regarded as a moderating variable in the relationship between resilience and self-efficacy, which reflects the psychological state of college students.

From the relationship between psychological resilience and self-efficacy, we can see that college students who were not afraid to face what happened after the pandemic had a higher sense of self-efficacy. They had more confidence in what they were doing because they were resilient to setbacks. Therefore, it is recommended that parents, teachers and school administrators stress the nurturing of resilience among young people.

At the same time, we found that higher creativity had a high impact on self-efficacy. If teachers taught college students to use creativity to adapt to challenges posed by the pandemic, they would help consolidate and strengthen the psychological resilience of students who would then have higher levels of self-efficacy that would be beneficial to their academic success and future career development. Therefore, parents, teachers and schools should pay attention to correct and positive guidance when exerting creative influence on college students, while being careful to avoid too much out-of-step thinking that can be counter-productive to the ability to adapt.

### 5.3 Limitations and future directions

There are some limitations in this study.

Firstly, as this is a cross-sectional study, we cannot infer cause and effect from the results or investigate the dynamic processes among the variables.

Secondly, all the participants were from one university. Due to the variations in the severity of outbreaks in different regions, the sample population was limited in representation, which may affect the validity of the results. Validity may also be affected by biases and influences such as social mobility.

Future researchers could use a longitudinal study design to enable long-term observation or expand the number and scope of participants, possibly selecting multiple data points from different college populations so as to make the results more generalizable. Future research could also address the optimization of the conceptual model. Another avenue of research is the exploration of other relevant moderating variables that may affect psychological resilience and self-efficacy.

## 6 Conclusion

We explored the relationships among the traits of psychological resilience, self-efficacy, and creativity in college students during the early stages of the COVID-19 pandemic. The results showed that resilience positively affected the self-efficacy and creativity of the participants. Creativity positively affected their self-efficacy as well. In addition, creativity moderated the relationship between resilience and self-efficacy. In other words, in the face of challenges posed by the COVID-19 pandemic (and possibly other crises), creativity is an important factor in enhancing college students’ self-efficacy. The findings of this study reveal that the psychological problems that college students with high self-efficacy may encounter in stressful and crisis situations should be viewed from a new perspective on resilience and self-efficacy.

## Data availability statement

The raw data supporting the conclusions of this article will be made available by the authors, without undue reservation.

## Ethics statement

The ethical approval was obtained from the Ethics Committee of Capital Normal University. The patients/participants provided their written informed consent to participate in this study.

## Author contributions

YX and GY designed the research. CY and JL carried out the literature review, data analysis, and wrote the manuscript. JZ was mainly responsible for data extraction and sorting. All authors have read and agreed to the submitted version of the manuscript.
